# Analysis of the outcome of liver transplantation patients in india - a prospective observational study

**DOI:** 10.1186/2197-425X-3-S1-A697

**Published:** 2015-10-01

**Authors:** S Jakkinaboina, M Varma, A Kakkar, P Peddapyata, M Aleem, S Paul

**Affiliations:** Apollo Health City, Critical Care Medicine, Hyderabad, India; Department of Hepatopancreatic Biliary Surgery, Apollo Health City, Hyderabad, India

## Introduction

Liver transplantation has become a widely accepted therapy for the management of the complications of cirrhosis and liver failure.

## Objectives

To analyze the patients undergoing the Liver transplant Recipients and the factors influencing the outcome of the patients at our tertiary care hospital.

## Methods

The patients underwent liver transplantation recepients between May 2014 to Jan 2015 were analysed. The data was collected from patient records after ethics committee approval. The data captured were Age,sex, ICU and hospital length of stay, bilirubin, albumin, INR, creatinine, intubation days, readmission,cold ischemia, warm ischemia, portal vein thrombosis, SGOT, SGPT, Vasopressors use, APACHE II, SOFA Score, MELD Score, arterial anatomy, graft complications, hospital and 60day mortality, readmissions, hospital acquired infections are monitored.

## Results

The total number of patients enrolled are 34. See tables 1, 2 and 3

**Table 1:** Demographic data of the transplant receipients.

Variable	Mean n (%)
Age in years	49.5
Sex Male	27 (79.41)
Cause of liver transplant (Alcohol cirrhosis)	20 (58.82)
APACHE II at admission	12.59
SOFA at admission	7
MELD Score	20.35
No Comorbidities	26 (76%)
INR	2.6
Total bilirubin mg/dl	4.51

**Table 2:** Outcome data of transplant patients.

Variable	Mean
ICU length of stay in days	4.85
Hospital length of stay in days	15.18
Cold ischemia time in minutes	243
Warm ischemia time in minutes	45.06
Ventilator days	0.85
Blood products received (PRBC)	9.41
Albumin mg/dl	2.91
Creatinine mg/dl	1.23
Max SGOT U/L / Max SGPT U/L / Lowest Platelets 10/mm	1227 / 592 / 42.3

**Table 3:** Mortality and other outcomes of the patients.

Variable	Number	Percentage
Graft Whole / partial	26 / 8	76.5 / 23.5
Mortality	1	2.94
Arterial anatomy abnormality	4	11.76
Renal Replacement therapy	1	2.94
Vasopressors use	5	14.71
Readmission	2	5.89
Portal Vein Thrombosis	2	5.89

The hospital mortality rate in our study is 2.94% (one patient).

All the patients are ABO compatable, 2 patients had early graft rejection of which one patient liver functions has completely recovered and the other had not fully recovered. The hospital acquired infections are nil in these patients.

26 (76.47%) liver donors are brain death patients. 5 (14.71%) receipient patients are HCV positive. The factors increasing the the mortality are Increased ICU and hospital length of stay, reintubation, readmission and renal replacement therapy.

The statistically significant factors which increase the ICU length of stay are increased Bilurubin, decreased Albumin, increased ventilator days, increased Cold Ischemia and Warm Ischemia time, use of Vasopressors, lowest Platelets and raised INR.

The statistically significant factors which increase the Hospital length of stay are decreased Albumin, increased ventilator days, reintubation, use of Vasopressors, readmission and lower platelets. The 60 day mortality is similar to hospital mortality.

## Conclusions

The patients undergoing the Liver transplant Recipients had good outcomes with less mortality and is comparable to the best centres in the world.

Hospital acquired infections are nil in these patients.

We need larger number to analyse the patients.
